# Visual stability—what is the problem?

**DOI:** 10.3389/fpsyg.2015.00958

**Published:** 2015-07-15

**Authors:** Andrew Glennerster

**Affiliations:** Department of Psychology, School of Psychology and Clinical Language Sciences, University of ReadingReading, UK

**Keywords:** 3D vision, depth perception, stereopsis, binocular vision, visual stability, moving observer, prediction error

The papers in this volume of *Frontiers* all relate to 3D vision. What, then, is a paper about visual stability doing among them? I will argue that these topics are really two sides of the same coin: if we are not to be surprised by the changes in images that occur as we move (we see a stable world), we must have some sort of representation of the 3D environment that has given rise to those images.

In general, 3D vision arises from viewing the world from multiple vantage points. Of course, 3D structure can be deduced from “pictorial cues” (i.e., those that are available from a single vantage point) but this is a special case relying on past experience. The more general case of multiple-view vision entails the construction of a coherent interpretation of changing images as the observer moves, based on the assumption of a static 3D scene. In other words, if an observer is not to be bewildered by these changing images, they must have a some type of internal representation that allows them to predict the way that the image will change as they move. This representation (whatever form it takes) is the basis of their 3D vision, while the successful predictions underlie their subjective impression of visual stability. Thus, 3D vision and visual stability are two different aspects of the same problem.

We can see this clearly when we look at the standard computer vision approach to 3D vision, whose goal is to find the 3-dimensional structure of the scene (*X*) and the rotation and translation of the camera in the *i*^th^ image (*R*_i,_ *T*_i_) that gives rise to a set of image locations (*x*_i_), where *x*_i_ is a 2 by *n* matrix of image locations for *n* points whose 3D world coordinates are stored in *X*. If *n* is sufficiently large, this has a closed form solution for two and three views (Hartley and Zisserman, 2000) and robust statistical methods have been established for solving the problem numerically for a larger number of frames (Triggs et al., 2000) and even in real time as the camera moves (Davison et al., 2007). In this case, the relationship between the problem of visual stability and 3D representation is very clear: stability implies that the set of 3D world coordinates (*X*) remains constant over time. However, there is no consensus that animals compute *X* or anything like it (Colby, 1998; Cheung et al., 2008; Pickup et al., 2013).

What *is* clear is that the brain predicts the sensory consequences of action all the time and, usually (perhaps always), this process does not rely on an explicit 3D reconstruction of the world. For example, output from the cortex to the spinal cord predominantly consists of:

- alpha motor signals that will lead to the contraction of muscles (a “force” command);
- gamma motor signals that provide a prediction of the length of the muscle (an “expected length” signal).

There are almost as many gamma-motor fibers innervating a muscle as there are alpha-motor fibers and, if sensory fibers from muscle spindles are included, the number sending a prediction of muscle length and monitoring the accuracy of the prediction far outweighs that involved in stimulating the contraction (McIntyre and Bizzi, 1993). In this non-visual domain, it is commonly assumed that the brain is capable of storing a long list of contexts in which different expected levels of resistance are likely to be encountered (hence different alpha/gamma co-activations). Rare counter-examples—like picking up an empty suitcase that we believe to be full—emphasize how often our predictions are correct. Storing a long list of expected contexts is very different from the notion of the brain carrying out active processes, such as receptive field “re-mapping,” that are often discussed in relation to visual stability (Duhamel et al., 1998; Ross et al., 2001; Wurtz, 2008; Melcher, 2011).

Others have provided a helpful critique of the notion that remapping of cortical receptive fields could explain the subjective phenomenon of visual stability (Cavanagh et al., 2010; Rolfs et al., 2011). They point out that many of the phenomena that have been interpreted as a remapping of receptive fields are compatible with a transfer of activation to a location that will be stimulated by a target after a saccade, so “priming” those neurons in advance of an imminent stimulus. It gives a head start to attentional processing at the retinal location where a target is about to appear and increases activation there.

It is a familiar idea that the role of cortical processing is to adapt to repetitive elements of the input signal and hence minimize the output sent from one level to the next. Even the output of photoreceptors, which adapt to mean luminance levels and signal only differences from this mean, fit this description; but cortical neurons can do the same trick in more than one dimension (e.g., Barlow and Földiák, 1989). “Top-down” feedback has an important long-term role in ensuring that the system remains tuned to the statistics of the sensory input and so it minimizes the “prediction errors” that are passed forward through the initial layers (e.g., Rao and Ballard, 1999). In theory, top-down feedback could also play a short-term role in interpreting the current image (e.g., Lee and Mumford, 2003). According to this view, perception (not just visual stability) is a process that attempts to match incoming “driving” signals with a “cascade of top-down predictions” (Clark, 2013). But it is also possible that visual stability is achieved without any active cascade of this type.

It is worth contrasting the active feedback processes that are proposed by authors such a Clark (2013), Friston (2010), and Lee and Mumford (2003) with those that have been discussed in relation to the perception of visual stability. The goals of these two types of “top down” signals are quite different: one is designed to reduce neural responses to expected, unsurprising stimuli while the other (e.g., Duhamel et al., 1998; Wurtz, 2008) has the effect of “priming” neurons in advance of a stimulus, with the effect that they respond more vigorously than otherwise.

It is clear that the example we began with, in which efferent gamma signals carry a prediction of expected muscle length, really is an active predictive process. But in this case, the brain has no option other than to export a prediction of muscle length, if movements are to be controlled smoothly at a spinal level. The same is not true of the task of predicting incoming visual data after a movement. In theory, this comparison could take place without any active “nulling.”

If there is no active process underlying visual stability, then, as I have mentioned, one possibility is that the brain stores a long list of contexts in which different expected visual input is likely to be encountered, much like the storage of expected resistances to muscular force discussed above. The cerebellum is well suited to this task as it can store large numbers of sensory contexts and associated motor commands (Marr, 1969; Albus, 1971; Apps and Garwicz, 2005). The idea is that sight, like other senses, is fundamentally based on sets of sensory contexts that are linked, through motor commands, to new, predicted sensory contexts. This has similarities to other proposals (e.g., Clark, 2013) but without invoking active “cascades” of feedback. Figure 1 illustrates this point in relation to 3D vision. The relevant movements in this case are rotational eye movements (saccades) and small translations (head movements) in different directions. An eye or camera is at the center of the sphere and can rotate through the angles indicated by the arcs on the sphere to fixate each of the objects in the scene. So, a sensory context (“I am looking at A”) plus a motivational context (“I want to be looking at B”) is sufficient to lead to a motor movement (camera rotation or saccade) that results in a new sensory context (“I am now looking at B”). A sphere, as shown in Figure 1, is a compact way to illustrate the spatial relationships but a neural instantiation might be more like a list of contexts, their associated motor outputs and expected sensory consequences. The sphere in Figure 1 also contains information about the depth structure of the scene and, again, these 3D relationships can be encoded as a list of sensorimotor contingencies. The details of this proposal can be found elsewhere (Glennerster et al., 2001, 2009) but the essence is that a representation can be stored that avoids 3D coordinates and yet contains sufficient information about the depth and direction of objects to be useful for predicting the sensory consequences of actions. Intuitively, one can see that if, as an observer moves their head around, they discover that the image of object B moves against a stable background (defined by the thin black arcs) then they have information that B is close whereas object A is distant. If the observer kept all the information about the motion parallax of A and B relative to other objects then they would, of course, have exactly the information needed to compute a 3D reconstruction of these objects in the scene. But if, on the other hand, the observer kept only a “summary” of the motion parallax information (e.g., for each arc in Figure 1, keep a running mean of the change in arc length computed over a series of observer translations) then the information stored is *not* the same. Because a representation of this type falls short of a full 3D reconstruction, some scene changes can be sneaked in “under the radar,” unnoticed by the observer; in these cases, the summary statistics of the image change are indistinguishable from those produced by a truly static scene. Proposals of this type should lead to experimentally testable predictions about the nature of visual representation of a 3D scene.

**Figure 1:**
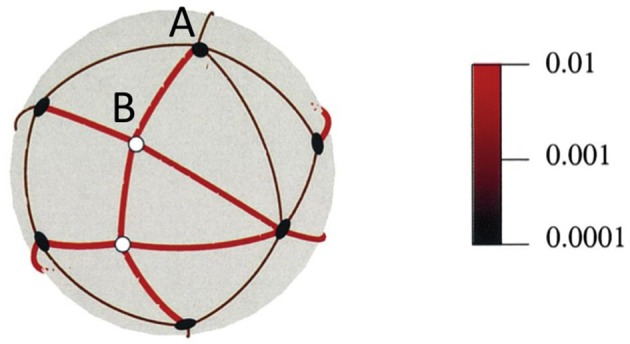
**An illustration of potential saccades between objects and how these change with head movements**. The eye is in the center of the sphere. The black circles show the visual directions of distant objects. The angles between these barely change despite head movements in 100 different random directions. By comparison, the angles between near objects (white circles) and other objects change to a much greater extent. The color scale shows mean angle change as a proportion of original angle for 100 translations of the same magnitude in different random directions. Reproduced, with permission, from Glennerster et al. (2001).

We perceive a stable world if, when we move our head and eyes, the image we receive falls within the set of images that we were expecting. The imprecision of our prediction means that sometimes we are fooled into thinking a scene is stable when actually it is not (Wallach, 1987; Jaekl et al., 2005; Tcheang et al., 2005; Glennerster et al., 2006). Demarcating the “equivalence classes” of scenes that can be interchanged and still perceived as stable is likely to be a useful tool in studying visual stability. But we need not suppose that visual stability *per se* is a particular problem. Predicting the sensory consequences of actions is ubiquitous in the brain and does not need to entail (nor is it normally considered to entail) complex “re-mapping.” We should not, without good reason, assume that visual stability has any special status over the problem of sensory prediction in other domains such as touch or proprioception and we should ask ourselves whether it really presents a tricky problem at all.

## Conflict of interest statement

The author declares that the research was conducted in the absence of any commercial or financial relationships that could be construed as a potential conflict of interest.
